# Facilitated Transport Membranes With Ionic Liquids for CO_2_ Separations

**DOI:** 10.3389/fchem.2020.00637

**Published:** 2020-08-18

**Authors:** Aidan Klemm, Yun-Yang Lee, Hongchao Mao, Burcu Gurkan

**Affiliations:** Department of Chemical and Biomolecular Engineering, Case Western Reserve University, Cleveland, OH, United States

**Keywords:** ionic liquid, facilitated transport membrane, carbon dioxide separation, aprotic heterocyclic anion, mixed matrix membrane, direct air capture

## Abstract

In recent years, significant development milestones have been reached in the areas of facilitated transport membranes and ionic liquids for CO_2_ separations, making the combination of these materials an incredibly promising technology platform for gas treatment processes, such as post-combustion and direct CO_2_ capture from air in buildings, submarines, and spacecraft. The developments in facilitated transport membranes involve consistently surpassing the Robeson upper bound for dense polymer membranes, demonstrating a high CO_2_ flux across the membrane while maintaining very high selectivity. This mini review focuses on the recent developments of facilitated transport membranes, in particular discussing the challenges and opportunities associated with the incorporation of ionic liquids as fixed and mobile carriers for separations of CO_2_ at low partial pressures (<1 atm).

## Introduction

To reduce CO_2_ emissions and mitigate the adverse effects of CO_2_-induced climate change (Ballantyne et al., 2018), removal of CO_2_, from atmosphere (Siriwardane et al., 2005) and pre-/post-process streams (Chen et al., 2012; Chen and Ho, 2016), has been a focus of research. The most common technologies to separate CO_2_ include adsorption (e.g., zeolites), absorption (e.g., liquid amines), and membranes in pre- and post-combustion CO_2_ capture. Pre-combustion capture is the removal of CO_2_ from pre-process gas mixtures (%CO_2_ > 20) such as syngas or biogas and typically involves separation pairs such as CO_2_/H_2_ and CO_2_/CH_4_, respectively. Post-combustion capture is the removal of CO_2_ from flue gas (5 < %CO_2_ <15) and typically involves a CO_2_/N_2_ separation pair. The energy demand is highest for adsorption and lowest for membrane separations. Zeolites are physisorption-based porous solid materials that are typically used in adsorption such as the removal of CO_2_ from air in spacecraft (Knox et al., 2017). Zeolites have high CO_2_ capacity, but suffer from extreme sensitivity to moisture (Chue et al., 1995; Cmarik and Knox, 2018). Membranes are energy-efficient, but struggle with the permeability/selectivity trade-off as described by Robeson (1991).

**Figure 1:**
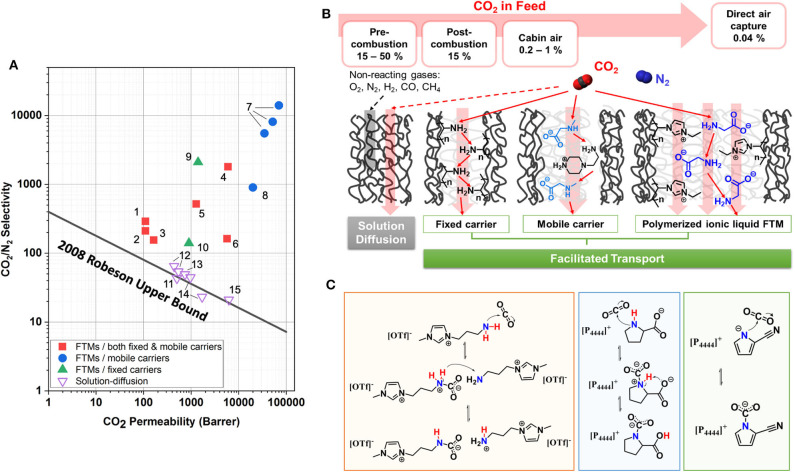
**(A)** FTMs with ILs in comparison to solution–diffusion membranes on Robeson plot for CO_2_/N_2_ separation. References for data: (1) (Chen and Ho, 2016), (2) (Chen et al., 2016), (3) (Han et al., 2018), (4) (Zou and Ho, 2006), (5) (El-Azzami and Grulke, 2009), (6) (Han et al., 2018), (7) (Moghadam et al., 2017a), (8) (Moghadam et al., 2017b), (9) (Kamio et al., 2020), (10) (McDanel et al., 2015), (11) (Teodoro et al., 2018), (12) (Tomé et al., 2018), (13) (Tomé et al., 2014), (14) (Scovazzo, 2009), (15) (Jindaratsamee et al., 2012). The selectivity and permeability data of FTMs shown in this figure are available in Table 1. **(B)** Schematics of CO_2_ transport in solution–diffusion membranes (feed with >15% CO_2_) and FTMs (feed with <15% CO_2_). Specific examples of polyvinylamine studied by Tong and Ho (2017) and amino acid salt by Chen and Ho (2016) illustrate the fixed carrier and mobile carrier FTMs, respectively. As an example to FTM with a polymerized IL, polymerized imidazolium with glycinate counter ion studied by Kamio et al. (2020) is also shown. **(C)** Reaction schemes for CO_2_-reactive ILs. Left: n-(3-aminopropyl)-n-methyl-imidazolium triflate. A zwitterion (${\text{CO}}_{2}^{\;-}$-${\text{NH}}_{2}^{\;+\_}$) forms by the formation of the CN bond in the first step, followed by the transfer of proton from the zwitterion complex to another amine, thus forming ammonium and carbamate ions. Middle: tetrabutyl phosphonium prolinate, [P_4444_][Pro]. Zwitterion formation in the first step is followed by an intramolecular hydrogen transfer that results in carbocylic acid. Right: tetrabutyl phosphonium 2-cyanopyrrolide, [P_4444_][2-CNpyr]. Nucleophilic addition of CO_2_ to AHA nitrogen forming carbamate with no hydrogen transfer involved.

The inherent limitation of polymeric membranes was defined in 1991, demonstrating the upper bound for the CO_2_/CH_4_ separation pair (Robeson, 1991). In 2008, Robeson redefined the upper bound in consideration of improvements in membrane technology. He also included CO_2_/H_2_ and CO_2_/N_2_ separations (Robeson, 2008). Most polymeric membranes operate on a pressure-driven solution–diffusion model and are limited in performance by the Robeson upper bound. Recently, facilitated transport membranes (FTMs) have been shown to surpass the Robeson upper bound. FTMs achieve high permeabilities without sacrificing selectivity, or vice versa. Figure 1A provides a perspective of FTMs in comparison to common membranes; this Robeson plot demonstrates the relation between CO_2_/N_2_ selectivity and CO_2_ permeability.

FTMs incorporate a reactive component that acts as a CO_2_ carrier, such as an amine-bearing polymer or a small molecule embedded within the polymer matrix. Earlier examples of FTMs based on polyamines and alkanolamines can be found in the review by Tong and Ho (2016). Very recent applications of FTMs include the CO_2_ capture from flue gas in pilot scale as demonstrated by Salim et al. (2018), Han et al. (2019), and Chen et al. (2020). The review here focuses on the incorporation of ionic liquids (ILs) to polymeric films. In particular, this review focuses on FTMs with ILs studied in the last 4 years, since the review by Tome and Marrucho (2016) on the IL-based materials for CO_2_ separations. ILs are versatile solvents with high CO_2_ solubilities that have been incorporated into a number of host materials. A brief background to FTMs and ILs is provided, followed by a review of the most recent FTMs with ILs either as fixed or mobile carriers, emphasizing the existing challenges and opportunities.

## Gas Transport Mechanism In FTMs

FTMs combine the selection capability of reactive processes with the reduced mass, volume, and energy advantages of membranes (Tong and Ho, 2016). The reactive component of the membrane, known as a carrier, reversibly reacts with CO_2_ to produce a CO_2_ carrier complex with its own concentration gradient across the membrane. At the permeate side, the CO_2_ complexation reaction is reversed as a result of low partial pressure of CO_2_ and the carrier is regenerated by releasing the captured CO_2_. Figure 1B illustrates the CO_2_ transport mechanism in FTMs in comparison to other polymeric membranes that achieve separations by solution–diffusion.

In solution–diffusion membranes, gas molecules diffuse through the free volume of the membrane that is created by the chain-to-chain spacing. The steady-state flux of CO_2_ is related to the segmental chain motion of the polymer and is expressed by Equation (1) (Zolandz and Fleming, 1992):

(1) $$\begin{array}{l} J_{CO_{2}}=\frac{D_{CO_{2}}\left(C_{CO_{2},f}-C_{CO_{2},p}\right)}{l} \end{array}$$

where *J*_*CO*_2__ is the steady-state CO_2_ flux, *D*_*CO*_2__ is the diffusion coefficient of CO_2_ in the membrane material, *C*_*CO*_2_,*f*_ and *C*_*CO*_2_,*p*_ are the feed and permeate CO_2_ concentrations, respectively, and *l* is the thickness of the membrane. For an FTM, an additional term is added to account for carrier-mediated transport of CO_2_ as in Equation (2) (Rea et al., 2019):

(2) $$\begin{array}{l} J_{CO_{2}}=\frac{D_{CO_{2}}\left(C_{CO_{2},f}-C_{CO_{2},p}\right)}{l}+\frac{D_{CO_{2}-C}\left(C_{CO_{2}-C,f}-C_{CO_{2}-C,p}\right)}{l} \end{array}$$

where *D*_*CO*_2_−*C*_ is the “effective diffusivity” modeling the combination of transmembrane CO_2_ complex diffusion and the CO_2_ hopping mechanism across the carriers. *C*_*CO*_2_−*C,f*_ and *C*_*CO*_2_−*C,p*_ are the CO_2_ complex concentration at the feed and permeate site, respectively. FTMs have two subgroups: mobile carrier and fixed carrier. For fixed carrier FTMs, the carrier is immobilized and the *D*_*CO*_2_−*C*_ only represents the hopping mechanism, where CO_2_ hops from one active site to another down the concentration gradient, as illustrated in Figure 1B (Cussler et al., 1989). For mobile carrier FTMs, the combination of Fickian (solution–diffusion mechanism), hopping, and complex diffusion (vehicular motion) pathways greatly enhances CO_2_ permeation as opposed to fixed carrier FTMs and conventional solution–diffusion membranes.

Permeability, *P*_*CO*_2__, of a membrane is determined by Equation (3) (Zolandz and Fleming, 1992):

(3) $$\begin{array}{l} P_{CO_{2}}=\frac{J_{CO_{2}}\times l}{\Delta p_{CO_{2}}}=S_{CO_{2}}\times D_{CO_{2}} \end{array}$$

where Δ*P*_*CO*_2__ is the pressure drop of CO_2_ across the membrane. *S*_*CO*_2__ is the solubility of CO_2_ in the membrane matrix that, along with diffusivity, governs the permeability of a membrane. With CO_2_ having the ability to complex with carriers, this additional chemical pathway greatly enhances the diffusivity and especially the solubility in FTMs in comparison with conventional solution–diffusion-based membranes. For thin films, and often for FTMs, the membrane thickness is difficult to define, and therefore, the permeance is often reported instead of permeability. Permeance is the flux of gas (i.e., CO_2_) per unit permeation driving force with units of GPU (gas permeation unit), equivalent to 1 × 10^−6^ cm^3^ (STP)·cm^−2^·s^−1^·(cm Hg)^−1^. Permeability has units of Barrers (1 Barrer = 1 GPU·μm).

Selectivity, α, is estimated by Equation (4) (Zolandz and Fleming, 1992):

(4) $$\begin{array}{l} \alpha_{ij}=\frac{P_{i}}{P_{j}} \end{array}$$

where *i* represents CO_2_ and *j* represents the other non-CO_2_ component of the separation pair.

## Developments Toward The Il-Based FTMs

ILs are salts that melt below 100°C. It is shown that increased alkyl chain length and fluorination significantly improve CO_2_ solubility in some ILs. The free volume of the liquid, originating from the weak anion–cation interactions and bulky structure, promotes CO_2_ solvation (Anthony et al., 2002). ILs are amenable to chemical functionalization to improve CO_2_ capacity. ILs with an amine-functionalized cation are reported to have CO_2_ capacities in the range of 0.5 mol CO_2_ per mol of IL (Bates et al., 2002). Most ILs with amino acid anions (AAs) (Ohno and Fukumoto, 2007; Gurkan B.E. et al., 2010) and aprotic heterocyclic anions (AHAs), (Gurkan B. et al., 2010) achieve equimolar CO_2_ capacities. More recently, dual functionalized ILs composed of diethylenetriamine cation and AHAs such as imidazolide, pyrazolide, and triazolide exceeded equimolar (~2 mol CO_2_ per mol IL), (Wu et al., 2019). Figure 1C illustrates the CO_2_ reactions with functionalized ILs. It should be emphasized that reaction enthalpy and most physical properties, not just the CO_2_ absorption capacity, can be tuned in ILs. Lastly, ILs have negligible volatility and higher thermal stabilities than molecular solvents. Therefore, ILs are considered promising alternatives to amines in absorptive CO_2_ separation, due to energy-efficient solvent regeneration, non-corrosivity, and high degradation temperature.

The main challenge using ILs to separate CO_2_ has been their high viscosity, usually caused by Coulombic interactions and hydrogen bonding. In this regard, the relatively low viscosity AHA ILs are the most promising, as they lack hydrogen bonding. Li et al. (2019) reported protic ILs with low viscosities (2–27 cP at 30°C) that achieve similar CO_2_ absorption capacities, especially in the presence of water. ILs have also been studied in the context of supported IL membranes (SILM). Cowan et al. (2016) and Bara et al. (2010) provide comprehensive reviews of SILMs for CO_2_ separations. ILs are especially advantageous for SILMs as they do not evaporate. However, the stability of SILMs under high transmembrane pressures remains to be a challenge as the IL may get pushed out of the micropores over time. A thicker membrane support (50–150 μm) is generally adopted to suppress this potential leakage; however, CO_2_ flux is significantly reduced due to the increased length of diffusion. One potential solution to this problem is to confine the IL media in nanopores, as the capillary force holding the ILs is high and far exceeding the pressure gradient imposed on the membrane. This resolves the leakage issue, and still renders a high CO_2_ flux through the membrane. Among various nanomaterials, graphene oxide (GO) nanosheets received great attention due to their high flexibility, good mechanical strength, and easy processability. Lin et al. (2019) confined a deep eutectic solvent that is selective to CO_2_, similar to ILs, into GO nanoslits as a highly CO_2_-philic GO-SILM. The group reported a structural change in the liquid that better promotes CO_2_ transport, even though the liquid is not reactive with CO_2_. This idea of ultrathin GO-SILMs greatly shortens the diffusion pathway of CO_2_ within the membrane, providing promise for the use of viscous ILs in membranes. Alternative strategies of utilizing ILs in membrane separations focused on polymer-IL composite, gelled IL, and polymerized IL membranes (Tome and Marrucho, 2016). A detailed review on the IL-based materials for CO_2_ separations by Tome and Marrucho (2016) discusses the prospects of these materials. The majority of these studies focus on CO_2_ separations for coal-fired power plants. In applications where CO_2_ needs to be separated from air, such as cabin air in submarines, spacecraft, or buildings, the partial pressure of CO_2_ is not sufficient for most of these membranes to efficiently perform. The only type of membrane that may meet the needs for such dilute separations are FTMs. Reactive ILs are promising to incorporate into FTMs because they provide tunable reaction chemistry and CO_2_ diffusivity with no vapor pressure.

### FTMs With Fixed Carriers

Polymers with CO_2_-reactive groups such as polyallylamine (Cai et al., 2007; Yegani et al., 2007; Zhao and Ho, 2012a; Prasad and Mandal, 2018), polyethyleneimine (Matsuyama et al., 1999; Xin et al., 2014; Yu et al., 2020), and poly(vinyl amine) (Qiao et al., 2015; Chen and Ho, 2016; Chen et al., 2016; Tong and Ho, 2017) have attracted particular attention as materials for FTMs. In fixed carrier FTMs, the reactive functional groups are anchored to the polymer backbone, which provides better structural integrity compared to FTMs with mobile carriers. In an effort to combine the high CO_2_ solubilities of ILs and improve the mechanical stability over SILMs, polymerized IL (PIL) membranes have been considered. Earlier examples of PILs demonstrated CO_2_/N_2_ selectivities comparable to SILMs, but with lower permeabilities. Several strategies improving CO_2_ transport in PILs include PIL/IL composites, PIL copolymers, and PIL/IL/inorganic particle mixed matrix membranes (MMMs). Out of these, MMMs are considered the most promising as they combine (i) the gas separation capability, (ii) thermal stability, and (iii) durability of inorganic filler materials with (iv) the good mechanical properties combined with (v) the processability of polymeric materials (Seoane et al., 2015; Tome and Marrucho, 2016).

Inorganic fillers such as zeolites (Shindo et al., 2014), hydrotalcite (Liao et al., 2014), mesoporous silica, and silica particles (Xing and Ho, 2011; Xin et al., 2016); organic fillers such as carboxylic acid nanogels (Li et al., 2015), polyaniline rods (Zhao et al., 2012, 2013; Li et al., 2015), carbon nanotubes (CNTs) (Deng and Hagg, 2014; Han et al., 2018), amine functionalized CNTs (Zhao et al., 2014), and graphene (Wang et al., 2016); and hybrid materials such as metal organic frameworks (MOFs) (Shen et al., 2016) and zeolitic imidazolate frameworks (ZIFs) (Zhao et al., 2015) have been used to date in MMMs and facilitated transport MMMs (FTMMMs) mainly for CO_2_/N_2_ and CO_2_/CH_4_ separations. The poor interfacial adhesion between fillers and polymers remains a challenge in this field, as this poor adhesion often results in gas percolation at defects, leading to a decrease in selectivity (Chung et al., 2007; Rezakazemi et al., 2014). Compared with inorganic fillers, hybrid porous materials such as MOFs and ZIFs that consist of metal ions or clusters and organic linkers show improved interfacial interaction with the polymeric matrix (Zhao et al., 2015).

Ma et al. (2016) reported three-component FTMMMs: (i) a porous MOF filler, NH_2_-MIL-101(Cr); (ii) a cation-functionalized reactive IL confined within the MOF; and (iii) polydioxane with intrinsic microporosity (PIM-1) as the matrix. With loading of the IL-filled MOFs at 5 wt.%, this novel fabrication led to excellent separation performance with a permeability of 2,979 Barrer and a CO_2_/N_2_ selectivity of 37 (Ma et al., 2016). Recently, Wang et al. (2020) fabricated FTMMMs from pyridine-based porous cationic polymers (PIPs) with Ac^−^, BF_4_^−^, and Cl^−^ anions as fillers in PIM-1. Owing to the π − π interactions between PIP and PIM-1, membranes with minimal defective voids were obtained. CO_2_ permeabilities in the order of 6,200 Barrer and CO_2_/N_2_ selectivities of 40 to 60 were measured. The purposes of ILs in MMMs are as follows: (1) to act as a glue, ensuring good adhesion between the filler and the polymer matrix; (2) to add tunability in CO_2_ affinity (solubility, diffusivity, and selectivity); and (3) to allow modulation of filler pore structure.

To date, the majority of the studies in CO_2_ separations with IL-incorporated membranes relied on the physical dissolution of CO_2_ in non-reactive ILs. Most recent examples include ionic polyimides that incorporate imidazolium-based ILs (Mittenthal et al., 2017). Szala-Bilnik et al. (2019, 2020) studied the impact of the anion in ionic polyimide–IL composite membranes, where the imidazolium functionality is present in both the polymer backbone and the plasticizer. They showed that the ion mobility in pure ILs does not translate to cationic membranes, due to ion coordination with the fixed cation. Nevertheless, the CO_2_ diffusivity in the membrane can still be tuned by the choice of the anion. Overall, ILs are exciting building blocks for polymeric membranes, with the promise of tunable separation performance for CO_2_ and even other target gas molecules.

In the context of FTMs, there are no examples for fixed carriers made of an amine-bearing polymerized IL cation. McDanel et al. (2015) reported an IL-based epoxy-amine ion gel FTM. However, the amine moiety was for crosslinking and required moisture to serve as a fixed carrier. Kamio et al. (2020) also reported PIL based FTM, where the counterion of the PIL, glycinate, is CO_2_ reactive as shown in Figure 1B. This is the first example to date of a fixed carrier made from a CO_2_-reactive PIL. These gel-type FTMs cannot be fabricated into stand-alone films due to the fragility of the gel, so a porous support or secondary gel network is used for mechanical support. In this study, the group used a new fabrication method that involves creating a gel suspension of the PIL and pressurizing the suspension through a hollow fiber support membrane. The solvent passes through the support layer, leaving behind a thin film of the reactive polymer around the inside of the hollow fiber support. This hollow fiber configuration is highly valuable in industrial applications due to its high membrane area-to-volume ratio. To prevent gel propagation into the pores of the support and clogging, Matsuyama and coworkers used dialysis to remove low-molecular-weight polymer and unreacted monomer from the gel suspension. Design and scalable fabrication of support membranes with pore structure that minimizes clogging remains an interest.

### FTMs With Mobile Carriers

Differing from FTMs with fixed carriers, the incorporation of reactive ILs as mobile carriers results in increased CO_2_ mobility due to both vehicular and hopping transport mechanisms (Doong, 2012). The idea of an IL mobile carrier was pioneered by Matsuyama et al. (1999) amid their developments in liquid absorber-based FTMs in the mid-1990's (Teramoto et al., 1996). They have been active in developing FTMs with liquid absorbers, such as aqueous amines (Teramoto et al., 1996), amino acid salts (Yegani et al., 2007), cation-functionalized ILs (Hanioka et al., 2008), AA ILs (Kasahara et al., 2012), and AHA ILs (Kasahara et al., 2014a; Otani et al., 2016). While the studied FTMs overcome the Robeson upper bound, the measured CO_2_ flux is limited by slow CO_2_ diffusion, a result of the high viscosity of the mobile carriers. Therefore, maintaining a reactivity–mobility balance of CO_2_ is crucial in designing the molecular structure of the mobile carrier.

Kasahara et al. (2014b) reported an IL-impregnated double-network ion gel membrane. One network of the ionomer gel immobilizes the IL mobile carrier, and the other provides mechanical support. These FTMs have stable separation performance with CO_2_ feed pressures as low as 0.1 kPa. However, the low diffusivity of CO_2_ necessitates operations under humid conditions and well above room temperature. While the high viscosity of the IL is advantageous against leaching and loss of the liquid, it hinders CO_2_ diffusivity (Moghadam et al., 2017a,b). Moghadam et al. (2017b) reported the highest CO_2_ permeability and selectivity to date in an AHA IL-based FTM ([P_2221O1_][Inda]): 20,000 Barrer and CO_2_/N_2_ selectivity of 900 under a feed of 2.5 kPa CO_2_ and 0% RH at 373K.

Otani et al. (2016) performed molecular dynamics simulations to predict the most effective AHA IL with a phosphonium cation for FTMs based on their viscosity. Preliminary calculations suggested that a pyrrolide or pyrazolide anion would improve transmembrane CO_2_ transport. However, the authors also emphasized that the anions have large CO_2_ binding energies, potentially hindering the desorption of CO_2_, which is a critical design parameter upon designing practical FTMs.

### FTMs With Both Mobile and Fixed Carriers

While there are limited examples of FTMs with both the mobile and fixed CO_2_ carriers, we are not aware of IL-based FTMs under this category. Studies by the Ho group are the only representatives of FTMs with both fixed and mobile carrier to the best of our knowledge. These thin-film composite membranes are made of polyallylamine fixed carrier and solid amine salts as the mobile carrier and studied for CO_2_ separation from flue gas (Zhao and Ho, 2012b; Chen and Ho, 2016; Chen et al., 2016; Han et al., 2018). High temperatures (>50°C) and humidity are essential factors for these membranes to facilitate transport of CO_2_, but these factors also promote penetration of the active layer into the support layer. To mitigate this, the Ho group incorporated high-molecular-weight polymers and multiwall carbon nanotubes (MWNTs) in their FTMs (Han et al., 2018). Such amine-bearing FTMs with both fixed and mobile carriers by the Ho group are among the very few FTMs that have been fabricated and tested at pilot scale (Salim et al., 2018; Han et al., 2019).

Table 1 summarizes the CO_2_ permeabilities and CO_2_/N_2_ selectivities of FTMs, specifically those with reactive ILs reported since 2015. It is suggested in both Figure 1A and Table 1 that FTMs with mobile carriers yield the highest permeability and selectivity, in comparison to FTMs with fixed carriers and FTMs with combined fixed and mobile carriers. While these FTMs were specifically designed for CO_2_ separation from flue gas, they are ideal platforms to work from for CO_2_ separation from dilute feed streams such as cabin air or atmospheric air. It is very likely that the next-generation FTMs will incorporate reactive ILs into the framework for performances like high permeability and selectivity.

**Table 1:** FTMs with superior CO_2_ permeabilities** (***P*_*CO*_2__) in units of Barrer and CO_2_/N_2_ selectivities (α) reported in the literature within 2015–2020, comparing FTMs with IL or PIL carrier components (shaded) to others with no IL.

**Membrane properties**			**Experimental conditions**				**Measured properties**		**References**
**FTM type**	** Composition**	**D (μm)**	**Feed CO_**2**_:N_**2**_**	**P_**feed**_ (bar)**	**T (^**°**^C)**	**RH**	**P_CO_2__ (Barrer)**	**α**	
Mobile carriers	DN gel/ [P_4444_][Pro]	150	0.1:99.9	1	30	30	35,000	5,500	Moghadam et al., 2017a
						70	52,000	8,100	
			0.05:99.95			70	70,000	14,000	
	DN gel/[P_2221O1_] [Inda]	80	2.5:97.5	1	100	0	20,000	900	Moghadam et al., 2017b
Fixed carriers	Poly([Veim] [Gly]) on PSf	4^a^	0.1:99.9	1	50	80	5600^*^ (1400)	2,100	Kamio et al., 2020
	Amine-crosslinked poly-[Im][TFSI] epoxy resin/[Emim] [DCA]	50	2.5:97.5	1.02	20	95	900	140	McDanel et al., 2015
Combined fixed and mobile carriers	PVAm/piperazine glycinate on PES	0.1	20:80	1.1	57	100	110^*^ (1100)	290	Chen and Ho, 2016
	PVAm/piperazine glycinate on zeolite Y on PES	0.1	20:80	1.1	57	100	110^*^ (1100)	210	Chen et al., 2016
	MWNT-reinforced PVAm/PZEA-Sar	0.17	20:80	1	57	100	166^*^ (975)	155	Han et al., 2018

The symbol

* *represents the permeabilities calculated from the reported permeance given in parenthesis in units of GPU*.

a *Value includes 3 μm diffusion resistance layer; PVAm, poly(vinylamine); PES, poly(ether sulfone); [PZEA][Sar], 2-(1-piperazinyl)ethylamine sarcosine; PTFE, poly(tetrafluoroethylene); DN, double network; [P_2221O1_][Inda], triethylmethoxymethyl phosphonium indazolide; [Veim][Gly], 1-vinyl-3-ethylimidazolium glycinate; PSf, poly(sulfone); [Im][TFSI], imidazolium bis(trifluoromethylsulfonyl) imide; [Emim][DCA], 1-ethyl-3-methylimidazolium dicyanamide*.

## Conclusions

FTMs have shown incredible promise for efficient gas separations at low CO_2_ partial pressures, with both mobile and fixed carriers achieving permeabilities and selectivities beyond the Robeson upper bound. The future of FTMs for CO_2_ separations from air is likely to involve incorporation of both fixed and mobile carriers simultaneously. The key takeaways from the reviewed literature on FTMs with ILs are summarized below:

- FTMs with liquid components like IL carriers generally give higher permeability and selectivity in contrast to their solid-based carrier FTM counterparts.
- When designing IL carriers, CO_2_ binding enthalpy is a critical property to tune, as it impacts the likelihood of CO_2_ desorption to regenerate the carrier. For mobile carriers, viscosity is also important, as most current FTMs are originally designed to function at high temperature and humidity, making them non-ideal for CO_2_ separations from air.
- For low CO_2_ partial pressure environments like cabin air, FTMs provide a promising technology platform, and perhaps the only type of membranes, to replace state-of-the-art zeolites for more efficient and continuous CO_2_ separations. However, FTMs still have a low CO_2_ flux at low partial pressures and struggle to process large volumes of gas. It will be important to study the amount of gas processable in practical timeframes, since this metric will highly depend on the CO_2_ diffusivity and hopping rate.

## Author Contributions

All authors listed have made a substantial, direct and intellectual contribution to the work, and approved it for publication.

## Conflict of Interest

The authors declare that the research was conducted in the absence of any commercial or financial relationships that could be construed as a potential conflict of interest.
